# The impact of polypharmacy on health outcomes in the aged: A retrospective cohort study

**DOI:** 10.1371/journal.pone.0317907

**Published:** 2025-02-03

**Authors:** Irene Boateng, Carlos Rodriguez Pascual, Paul Grassby, Zahid Asghar, Kinda Ibrahim

**Affiliations:** 1 School of Pharmacy, College of Science, University of Lincoln, Lincoln, United Kingdom; 2 Academic Geriatric Medicine and National Institute for Health Research Applied Research Collaboration (ARC) Wessex, University of Southampton, Southampton, United Kingdom; Gabriele d’Annunzio University of Chieti and Pescara: Universita degli Studi Gabriele d’Annunzio Chieti Pescara, ITALY

## Abstract

**Objectives:**

To estimate the prevalence of polypharmacy among community-dwelling adults in the UK and determine its association with mortality, hospitalization, adverse drug reactions and falls at one and five years. To also determine the effect of polypharmacy on the outcomes in different patient groups.

**Methods:**

A retrospective cohort study was carried out using 1000 patients aged 75 years and above from the Clinical Practice Research Datalink. The study periods for the one- and five-years analysis were January 2010-December 2010 and January 2010-December 2014 respectively. Sociodemographic and clinical variables were retrieved using medical and product codes. Polypharmacy was defined as the use of five or more medicines. The association between polypharmacy and mortality, falls, adverse drug reactions, or hospitalization was determined using cox regression analysis while confounding for age, sex, Charlson’s comorbidity index, potentially inappropriate medicines, hospitalization prior to study, and falls prior to study. Subgroup analysis was used to determine the effect of polypharmacy on the outcomes for different patient groups.

**Key findings:**

977 people were reviewed. 36% were male and the mean age was 83 years. The prevalence of polypharmacy was 47%. Adjusted hazard ratios with their 95% confidence intervals for association between polypharmacy and outcomes at five years were: mortality 1.60 (1.30–2.00), hospitalization 1.49 (1.30–1.70), falls 1.49 (0.90–2.40) and adverse drug reactions 0.97 (0.50–1.80). The results for the one-year analysis were mortality 2.37 (1.40–3.90), hospitalization 2.47 (1.40–4.30), and falls 0.37 (0.03–4.00).

**Conclusion:**

Polypharmacy was found to be a risk factor for mortality and hospitalization. The risk increased with an increase in age, potentially inappropriate medicines and comorbidities.

## 1. Introduction

Polypharmacy is commonly defined as the use of five or more medicines [1]. Its prevalence among over 65-year-olds has been estimated between 17–52% [2–5]. Medication non-adherence, cognitive impairment and Adverse Drug Reactions (ADRs) are associated with polypharmacy [6]. The risk associated with ADRs increases as the number of medicines increase [6]. Older patients are particularly susceptible to ADRs due to multiple comorbidities, a high prevalence of multiple medications and age-related changes in pharmacokinetics and pharmacodynamics activities [6].

Most of the research into polypharmacy has been done in over 65-year-olds and have sought to establish its relationship with sociodemographic and clinical factors [2–4]. The findings have been inconsistent and in some comorbidity was poorly adjusted or not adjusted for [3,5,7]. Participants from single centre settings were used by others [8–9] and the short- and long-term effects of polypharmacy and mortality has only been estimated by one study [10]. Misclassification of polypharmacy was also possible in some studies as it was only estimated at baseline [10–12]. This poses a problem as new medicines can be prescribed, or existing medicines reduced during the follow up period. This study aims to address these gaps by estimating polypharmacy as the average number of medicines and determining its association with mortality, falls, ADRs and hospitalizations at one and five years in over 75-year-olds. A population database will be used, and polypharmacy will be defined as the use of five or more medicines. This definition was chosen based on previous studies and recommendation by the World Health Organisation [1].

## 2. Method

### 2.1. Study design

This was a retrospective cohort study to determine the association between polypharmacy and mortality, falls, hospitalizations and ADRs at one and five years using proprietary data from the Clinical Practice Research Datalink (CPRD). The terms and conditions of use meant that the study periods had to be from January 2010-December 2010 and January 2010-December 2014 for the one- and five-years analysis respectively. The data are still relevant as it reflects current prescribing practices and the ongoing challenge of multimorbidity in the aged population.

The CPRD contains about 14.2 million records making it the world’s largest data of anonymized longitudinal patients records. It collects patient data from a network of General Practitioner practices across the UK. Fifty-eight percent of the data are linked to a range of other health-related datasets such as the Hospital Episode Statistics (HES), cancer registry data and mortality data from the Office for National Statistics (ONS). This provides a longitudinal, representative UK population health dataset which encompasses 45 million patients, including 13 million currently registered. For more than 30 years, research using CPRD data has informed clinical guidance and best practice, resulting in over 2400 peer-reviewed publications investigating drug safety, use of medicines, effectiveness of health policy, healthcare delivery and disease risk factors [13].

### 2.2. Sample size

Six hundred subjects were needed to detect a prevalence of polypharmacy of 50% with 4% precision and 95% confidence. Also based on an estimated five-year mortality rate of 20% in patients without polypharmacy and a 38% increased risk (RR  =  1.38) in global mortality in patients with polypharmacy, 492 patients were included in each group with 80% power, two-sided P <  0.05 [14]. The final sample size for the study was 1000 patients.

#### 2.2.1. Participants.

The participants were a random sample of 1000 primary care adults registered in the CPRD before 1st January 2010 who were 75 years old and above, in whom the number and name of prescribed drugs and five-year outcomes were available, and whose registry were linked with the HES and the ONS. The patients were required to have three months of lead in data to ensure the long-term use of medicines. Terminally ill patients or those in palliative care were excluded.

### 2.3. Variables

#### 2.3.1. Exposure variable.

**Polypharmacy:** The number of average prescribed medicines was estimated by adding all drug prescription days at one or five years and dividing the sum by the follow up periods which were 365 days or 1825 days for one and five years respectively or lesser. Drug prescription days was estimated by dividing the quantities of medicines prescribed by the daily dose. This method has been used by Park et al [15]. Average prescribed medicines of < 5 indicated no polypharmacy and ≥ 5 indicated polypharmacy. Medicines for single use, e.g., short courses of antibiotics were excluded.

#### 2.3.2. Independent variables.

Charlson’s Co-morbidity Index was used as an indicator of co-morbidity. It consists of 17 different categories of diseases assigned different weights based on their prediction of all-cause mortality in one year. It is widely used, and its validity has been confirmed by comparison with other indices [16]. The index was applied to the data using product codes and International Classification of Diseases (ICD)-10 codes for the various components. For the regression analysis, comorbidity was categorized into no morbidities (0) and one or more morbidities (≥1). Age, sex, and gender variables were retrieved from the patient file. Potentially Inappropriate Medicines (PIMs) were estimated using the Screening Tool for Older Peoples Prescription (STOPP) criteria version 2. The STOPP criteria is organized according to physiological systems and the version 2 contains 114 criteria. It has global relevance as it has been used in several countries to detect PIMs and improve patient outcomes [17]. Falls prior to study was defined as the presence of ICD-10 codes for falls (S1 File) from 1st January 2009 to 31st December 2009. Hospitalization prior to study was estimated using hospitalization data from the HES and applying dates from 1st January 2009 to 31st December 2009.

#### 2.3.3. Outcome variables.

Falls and ADRs were defined as the presence of ICD-10 codes for falls and ADRs respectively (S1 File). Mortality and hospitalization data were present as linked files from the ONS and HES respectively.

### 2.4. Statistical analysis

All variables were retrieved and analysed using Stata version 15. Frequencies and means were used to describe qualitative and quantitative variables respectively. Outcome variables were defined as binary i.e., death or alive, hospitalized or not, presence or absence of ADRs and falls. Relationship between polypharmacy and outcomes were estimated using multivariate cox regression analysis, confounding for age, sex, morbidity, PIMs, hospitalization prior to study and falls prior to study. The results were summarised as Hazard Ratios (HR) with their 95% confidence interval (S2 File). Subgroup analyses were carried out for outcomes which showed positive associations with polypharmacy by grouping the data into different categories of gender, age, PIMs and comorbidities (S2 File)

### 2.5. Ethics

The protocol for this study was approved by the Independent Scientific Advisory Committee of the Medicines and Healthcare Regulatory Agency (MHRA) UK.

## 3. Results

Data of 1000 people were received from the CPRD, but the data of 977 people were analysed as 23 people had missing data on drug use. 36% were male, the mean age was 83 years, and the mean comorbidities recorded was 2.18. 70% were prescribed PIMs, 30% were previously hospitalized and 2.5% had previous falls. Polypharmacy was present in 457 people representing 47% (Table 1).

**Table 1:** Baseline characteristics of sample.

	With polypharmacy	Without polypharmacy	Value	Total
	N = 457	N = 520		N = 977
Age, mean ± SD	83.24 ± 5.43	82.94 ± 5.50	0.90	83 ± 5.52
Gender				
Male N (%)	161 (35.23)	190 (36.54)	0.67	351 (35.90)
Female N (%)	296 (64.77)	330 (63.46)		626 (64.10)
Hospitalization prior to study N (%)	165 (36.11)	132 (25.38)	P < 0.01^b^	297 (30.40)
Falls prior to study N (%)	16 (3.50)	9 (1.73)	0.80^b^	25 (2.56)
Potentially inappropriate medicines N (%)	356 (77.90)	341 (65.58)	P < 0.01^b^	697 (69.91)
Charlton’s Comorbidity Index, mean ± SD	2.56 ± 2.05	1.81 ± 1.82	P < 0.01^a^	2.18 ± 1.94
One year analysis				
Mortality	42 (9.20)	28 (5.38)	P < 0.01^b^	70 (7.16)
Hospitalization	37 (8.10)	23 (4.42)	P < 0.01^b^	60 (6.14)
Falls	1 (0.22)	3 (0.58)	0.38^b^	4 (0.41)
Adverse drug reactions	1 (0.22)	0 (0.00)	0.29^b^	1 (0.22)
Five years analysis				
Mortality	190 (41.57)	166 (31.90)	P < 0.01^b^	356 (36.44)
Hospitalization	404 (88.40)	378 (72.69)	P < 0.01^b^	782 (80.04)
Falls	41 (8.97)	30 (5.77)	0.05^b^	71 (7.27)
Adverse drug reactions	27 (5.90)	22 (4.23)	0.23^b^	47 (4.81)

N =  total number; SD, standard deviation.

^a^Mann- Whitney U test statistic.

^b^Chi square statistic.

At one year, people with polypharmacy had more deaths (9.20% vs 5.38%) and hospitalizations (8.10% vs 4.42%) than those without (Table 1). At five years, people with polypharmacy had more deaths (41.57% vs 31.90%), hospitalizations (88.40% vs 72.69%), falls (8.97% vs 5.77%) and ADRs (5.90% vs 4.23%) than those without (Table 1).

### 3.1. Relationship between polypharmacy and outcomes

#### 3.1.1. Mortality.

Polypharmacy was positively associated with mortality at one and five years. The unadjusted hazard ratios at one and five years were 1.75 (1.10–2.80) and 1.47 (1.20–1.80) respectively. Adjusted hazard ratios were 2.37 (1.40–3.90) and 1.60 (1.30–2.00) for one and five years respectively (Table 2).

**Table 2:** Effect of polypharmacy on outcomes.

One Year Analysis					Five Years Analysis			
	Unadjusted HR with 95% Confidence Interval (CI)	P value	Adjusted HR with 95% CI	P value	Unadjusted HR with 95% CI	P value	Adjusted HR with 95% CI	P value
Mortality	1.75 (1.10–2.80)	P < 0.01	2.37 (1.40–3.90)	P < 0.01	1.47 (1.20–1.80)	P < 0.01	1.60 (1.30–2.00)	P < 0.01
Hospitalization	1.95 (1.10–3.40)	P < 0.01	2.47 (1.40–4.30)	P < 0.01	1.62 (1.40–1.90)	P < 0.01	1.49 (1.30–1.70)	P < 0.01
Falls	0.38 (0.04–3.70)	0.41	0.37 (0.03–4.00)	0.41	1.64 (1.00–2.60)	0.05	1.49 (0.90–2.40)	0.11
Adverse drug reactions	Omitted		Omitted		1.39 (0.80–2.40)	0.49	0.97 (0.50–1.80)	0.93

Models adjusted for age, gender, PIMs, comorbidity, falls prior to study and hospitalization prior to study, HR, hazard ratio, CI confidence interval.

#### 3.1.2. Hospitalization.

Polypharmacy was positively associated with hospitalization at one and five years. The unadjusted hazard ratios at one and five years were 1.95 (1.10–3.40) and 1.62 (1.40–1.90) respectively. The adjusted hazard ratios were 2.47 (1.40–4.30) and 1.49 (1.30–1.70) for one and five years respectively (Table 2).

#### 3.1.3. Falls.

Polypharmacy was not associated with falls at one or five years. The unadjusted hazard ratios at one and five years were 0.38 (0.04–3.70) and 1.64 (1.00–2.60) respectively. The adjusted hazard ratios were 0.37 (0.03–4.00) and 1.49 (0.90–2.40) for one and five years respectively (Table 2).

#### 3.1.4. ADRs.

Polypharmacy was not associated with ADRs at five years. The unadjusted and adjusted hazard ratios were 1.39 (0.80–2.40) and 0.97 (0.50–1.80) respectively (Table 2). Full results are available in S2 File.

### 3.2. Subgroup analysis

The effect of polypharmacy on mortality was high in women than men (OR 1.89 vs 1.73), in participants aged 75–85 years than 86 years and above(OR 1.77 vs 1.73), in those with ≥ 6 PIMs than those with less (OR 1.79 vs 1.64), and in those with 3-6 morbidities than 1–2 morbidities (OR 3.55 vs 1.67) (Table 3).

**Table 3:** Relationship between polypharmacy and mortality or hospitalizations for specific patient groups.

Participant groups	Outcomes			
	Mortality		Hospitalization	
	Adjusted Odd Ratio (OR) (95% CI)	P value	Adjusted OR (95% CI)	P value
Women	1.89 (1.29–2.78)	P < 0.01	2.44 (1.46–4.09)	P < 0.01
Men	1.73 (1.05–2.86)	P < 0.01	1.47 (0.77–2.81)	0.25
75–85 years	1.77 (1.21–2.57)	P < 0.01	1.69 (1.05–2.72)	P < 0.01
≥86 years	1.73 (1.06–2.08)	P < 0.01	3.08 (1.42–6.67)	P < 0.01
≤5 PIMs	1.64 (1.17–2.29)	P < 0.01	2.19 (1.39–3.43)	P < 0.01
≥6 PIMs	1.79 (1.34–2.41)	P < 0.01	2.35 (1.59–3.46)	P < 0.01
1–2 morbidities	1.67 (1.19–2.36)	P < 0.01	1.87 (1.19–2.93)	P < 0.01
3–6 morbidities	3.55 (1.71–7.38)	P < 0.01	2.35 (0.94–5.84)	0.07

The effect of polypharmacy on hospitalization was high in women than men (OR 2.44 vs 1.47), in participants aged 86 years and above than 75–85 years group (OR 3.08 vs 1.69), in those with ≥ 6 PIMs than those with less (OR 2.35 vs 2.19), and in those with 36 morbidities than 1–2 morbidities (OR 2.35 vs 1.87). The result for men and people with 3–6 morbidities was statistically insignificant (Table 3). Full results are available in S2 File.

**Table 4:** Effect of interactions between polypharmacy and different groups on outcomes.

Interactions	Outcomes			
	Mortality		Hospitalization	
	Adjusted OR (95% CI)	P value	Adjusted OR (95% CI)	P value
Polypharmacy and 75–85 years group	1.89 (1.31–2.74)	P < 0.01	1.82 (1.14–2.91)	P < 0.01
No polypharmacy and ≥ 86 years group	3.60 (2.51–5.16)	P < 0.01	1.19 (0.79–1.79)	0.38
Polypharmacy and ≥ 86 years group	5.88 (3.77–9.17)	P < 0.01	3.01 (1.45–6.09)	P < 0.01
No polypharmacy and female	0.87 (0.61–1.26)	0.48	0.97 (0.66–1.43)	0.90
Polypharmacy and female	1.63 (1.07–2.47)	P < 0.01	2.22 (1.27–3.89)	P < 0.01
Polypharmacy and male	1.69 (1.05–2.72)	P < 0.01	1.62 (0.86–3.02)	0.13
Polypharmacy and ≤ 5 PIMs	1.83 (1.36–2.45)	P < 0.01	2.15 (1.45–3.16)	P < 0.01
Polypharmacy and ≥ 6 PIMs	1.22 (0.54–2.75)	0.63	omitted	
No polypharmacy and ≥ 6 PIMs	1.93 (0.52–7.61)	0.32	1.18 (0.13–10.41)	0.88
Polypharmacy and ≤ 2 morbidities	1.81 (1.35–2.44)	P < 0.01	2.06 (1.38–3.06)	P < 0.01
No polypharmacy and 3–6 morbidities	6.05 (0.50–72.80)	0.16	0.90 (0.08–10.07)	0.93

## 4. Discussion

This study determined the long- and short-term effects of polypharmacy on mortality, falls, ADRs and hospitalizations, the effect of polypharmacy on the outcomes in different patient groups, and the interactions between polypharmacy and different groups on the outcomes (Table 4). The main finding was that polypharmacy is a risk factor for mortality and hospitalization in the short and long term; the risk associated with it are high in women, people aged ≥ 86 years, people with six or more PIMs, and those with 3–6 morbidities.

### 4.1. Strengths and limitations

This study used data from the CPRD which is a very large anonymised primary care record covering most of the primary care practices in the UK. The variables were measured accurately using valid methods. The participants were 75 years old and above providing information on this group of population where little is known about medication related outcomes [18]. The database did not provide information on over-the-counter medicines, and we do not know if the prescribed medicines were taken by the patients. This has the potential to underestimate or overestimate the prevalence of polypharmacy. It was also not possible to adjust for all possible confounders that could affect the outcomes, e.g., smoking status, social determinants of health, frailty or disability related to mortality or hospitalization.

### 4.2. Comparison with other studies

Evidence suggests that polypharmacy is a risk factor for hospitalization and mortality in the aged with the risk increasing with an increase in age, PIMs and comorbidities [3,10,11,19–21]. This is because polypharmacy increases the risk of ADRs which can eventually lead to hospitalization and death. Hospitalization can lead to new diagnosis which may require further drugs, or that old therapies need to be replaced by new or more complex ones [22]. These can potentially increase the number of PIMs. The ageing process is also associated with changes in pharmacokinetic and pharmacodynamic activities which predisposes the aged to ADRs. Furthermore, increasing comorbidity or disease severity can lead to polypharmacy or ADRs, though the primary mortality or hospitalization risk might also be due to the severity of illness and not polypharmacy. In the above studies, the participants were aged 65 years and above whereas we used people aged 75 years and above. Polypharmacy was estimated as the cumulative number of medicines whereas we estimated it as the average number of medicines. Some couldn’t adjust for morbidity [3] whereas we did and only one study looked at the short- and long-term effect of polypharmacy on the outcomes [10].

For both outcomes, the risk was higher at one year than five years. The reason for this was that as the data was presented, there was a higher proportion of death in people with one year follow up than those with five years follow up. Also, the effect of polypharmacy at five years was not a composite effect of one to four years. Richardson and colleagues also found the association between polypharmacy and mortality to be highest in the short term than the long term [10]. In contrast, some studies have not found polypharmacy to be a risk factor for mortality in the aged (≥65 years) [9,23]. Mortality rates of 5%–9% were reported whereas the mortality rates for this study were 9.20% and 42% at one and five years respectively [9]. Sganga et al. defined polypharmacy as the use of eight or more medicines [9] whilst a definition of five or more medicines was used in this research. Schottker and colleagues in addition to adjusting for clinical and sociodemographic factors, further applied a propensity score which measures an individual’s tendency towards polypharmacy [23].

In contrast to other studies [24,25], this study did not find a positive relationship between polypharmacy and ADRs or falls. In fact, the result for ADR at one year was omitted. This was due to the inadequate recording of ADRs and falls in the CPRD database. Only one ADR was recorded in our sample at one year. Hypoglycaemia did not also have a significant effect on falls when included in the model. The CPRD depends on doctors to record ADRs and it is known that only 3%–13% of ADRs are recorded by medical staff and doctors [26].

### 4.3. Implications for research and practice

Polypharmacy is well recognized by policy makers. It is incorporated in the UK combined model for predicting hospital admission and the US resident assessment instrument minimum dataset [27]. National guidance on managing polypharmacy has also been published in Scotland and there is an increasing realization that clinical guidelines, which are currently designed for single disease conditions, should address the clinical complexities of multimorbidity [27].

These notwithstanding, the prevalence of polypharmacy among the aged is high. Although the use of many medicines is not necessarily bad and should not be misinterpreted as a characteristic of care that inevitably leads to adverse outcomes [27], it is associated with riskier prescribing and is often a problem in people who are physically frail or have cognitive impairment [28]. Its consequences must be addressed, and this can be done in part, through continued medical education and clinical guidelines, particularly for common conditions that affects older patients [29]. Single disease guidelines do not consider the life expectancy of the aged; therefore, it does not recommend the stoppage of chronic or preventative medicines. Clinical guidelines that will initiate and stop treatments based on an individual’s life expectancy must be encouraged [28]. Tools need to be developed to identify individuals who are likely to be at risk from polypharmacy. Specifically, these tools should target women, the aged, people with a high number of morbidities and PIMs, and in such people, interventions that have the potential to reduce it must be applied. One of such intervention is medication review. It has been known to increase concordance and drug appropriateness, unplanned hospitalization and mortality [30]. Polypharmacy is a risk factor for mortality and hospitalization. Knowing this is important for clinical care and service planning. At an individual level, an assessment of the risk of death and hospitalization can inform decisions on preventive care [31]. Also, deprescribing an intervention that can be used to reduce the intake of unnecessary medicines can be a focus in the care of the aged as the effectiveness and safety of medicines can change with increasing age [32].

The results of this research also indicate that the CPRD must actively encourage health care workers to adequately record events, especially ADRs. Alternatively, they could link their database with pharmacovigilance data from the yellow card scheme so enough information on ADRs is retrieved for research purposes. The yellow card scheme allows patients to report any suspected ADRs to the MHRA. Patient reports contain a higher number of suspected ADRs, are usually richer in their descriptions than those from health care professionals and do consider the effects of ADRs on their lives [33].

## 5. Conclusion

The prevalence of polypharmacy in over 75-year-olds in the UK is high. Polypharmacy is a risk factor for mortality and hospitalizations in the short and long term. The risk increases with an increase in age, comorbidities, PIMs, and in women. Falls and ADRs were not associated with polypharmacy due to the inadequate recordings of these events in the CPRD database. Research into the management of inappropriate polypharmacy must be encouraged and the CPRD must put in interventions to ensure the adequate recording of falls and ADRs in their databases.

## Supporting information

S1 File ICD 10-CODES. (DOCX)

S2 File Results of regression analysis. (DOCX)
