# Supplementary material for: The impact of polypharmacy on health outcomes in the aged: A retrospective cohort study
Source: PLoS One. 2025 Feb 3;20(2):e0317907. doi: 10.1371/journal.pone.0317907 (PMC11790100; doi:10.1371/journal.pone.0317907)
Supplement: S1 File — (DOCX) [file pone.0317907.s001.docx]

**S1: ICD10 – CODES**

Falls: 44119 109088 98382 32737 55743 58988 46559 42670 7970 26292 8730 6815 6835 6008 8694 105499 66395 107609 48309 96951

Adverse drug reactions : 43949 66407 97301 30491 30814 25269 61507 48600 43814 58118 7252 42993 45916 48350 48622 55033 21660 96808 47857 39450 94225 63441 42754 36211 13400 98477 104802 93338 42311 59874 20697 38170 35881 45584 16819 105774 70518 68285 55949 96532 96151 73787 33660 37746 42826 21039 61685 24593 9752034461 70072 20388 36545 34768 306 59450 13367 49120 38142 61941 37362 46076 21451 44921 98766 108442 100033 63364 65684 66689 22952 44510 73976 66724 100837 98426 99902 47209 52649 49686 18396 26107 43945 99224 45437 48655 20916 94878 48619 57782 50174 58674 94112 99823 55491 73835 73877 48620 13395 70434 48634 89713 48698 72205 57383 13390 50949 110476 56181 37058 30134 20747 51116 64864 47141 22305 55631 45023 68465 26495 48688 48640 13026 60010 55032 9998 13388 44602 63165 20513 26484 51840 63207 4769 46286 103633 56400 42992 110734 21517 49689 95626 111087 21920 38171 62724 73837 31529 55700 24004 26487 94058 73889 68467 73867 73297 49685 63642 108617 47248 67690 103415 97026 73863 63655 73872 94711 55910 66841 26482 49680 42096 48621 46411 94266 61292 48591 46541 96531 52662 94634 63649 51064 73973 111569 43499 55948 45021 48112 65676 31899 217575 5780 97190 73875 9135 5717 35480 72756 34470 73852 48672 48676 30846 57147 21658 13025 108026 94772 98212 36535 51963 6913 20451 48668 68306 48779 47589 112367 46020 68335 46796 55126 44040 41413 42804 31674 48627 35478 48629 43947 48674 13034 65428 21576 39564 51647 47372 55695 21928 30039 34098 13393 32761 73869 13405 73887 67493 69347 47382 13385 73857 51063 39720 56225 51430 89735 73880 47823 67266 71515 42994 59549 73832 57356 13044 62593 48979 68928 53534 7253 38866 55136 89738 94775 73882 94771 46275 112252 51289 50038 65578 63877 54457 68334 94722 67051 48614 60117 58985 45022 110946 13031 102257 44531 94109 26492 60768 13029 57521 71958 47824 68406 70321 98985 73864 100376 71318 107269 73849 30770 63233 96182 59697 56401 97183 50578 44350 73883 50098 61210 97896 55816 47357 16392 41409 11667 13010 56471 66984 43946 58907 40611 28027 39808 48599 48615 99186 60129 5483 30752 49864 41323 61975 21705 101133 48136 40864 50176 7569 109335 49679 73878 48351 41412 32384 93913 90509 49678 55911 69395 60374 73844 13030 59545 20497 48604 19616 92893 42451 34468 63307 32394 111421 54953 20067 48776 73855 48976 96388 68337 63019 111856 60310 57289 26485 56474 60240 59485 381 73862 6762 59633 91665 59002 73851
