# Supplementary material for: The impact of polypharmacy on health outcomes in the aged: A retrospective cohort study
Source: PLoS One. 2025 Feb 3;20(2):e0317907. doi: 10.1371/journal.pone.0317907 (PMC11790100; doi:10.1371/journal.pone.0317907)
Supplement: S2 File — (DOCX) [file pone.0317907.s002.docx]

**S2: Result of regression analysis**

Table 1: Relationship between polypharmacy and mortality at one and five years expressed as Hazard Ratios (HR) with 95% confidence interval

|  | **ONE YEAR** | | | | **FIVE YEARS** | | | |
| --- | --- | --- | --- | --- | --- | --- | --- | --- |
|  | **Univariate** | | **Multivariate** | | **Univariate** | | **Multivariate** | |
|  | **HR 95% CI** | **P value** | **HR 95% CI** | **P value** | **HR 95% CI** | **P value** | **HR 95% CI** | **P value** |
| **Polypharmacy** |  |  |  |  |  |  |  |  |
| No(reference) |  |  |  |  |  |  |  |  |
| Yes | 1.75(1.1-2.8) | P<0.05 | 2.37(1.4-3.9) | P<0.05 | 1.47(1.2-1.8) | P<0.05 | 1.60(1.3-2.0) | P<0.05 |
| **Age** | 1.10(1.1-1.2) | P<0.05 | 1.10(1.0-1.1) | P<0.05 | 1.12(1.0-1.2) | P<0.05 | 1.13(1.1-1.2) | P<0.05 |
| **Gender** |  |  |  |  |  |  |  |  |
| Female(reference) | | | | | | | | |
| Male | 0.87(0.5-1.4) | 0.58 | 0.95(0.6-1.6) | 0.85 | 0.96(0.8-1.2) | 0.70 | 1.10(0.9-1.4) | 0.39 |
| **Previous Hospitalization** | | | | | | | | |
| No(reference) |  | | | | | | | |
| Yes | 1.76(1.1-2.8) | P<0.05 | 1.76(1.1-2.8) | P<0.05 | 1.39(1.1-1.7) | P<0.05 | 1.34(1.1-1.7) | P<0.05 |
| **Previous Falls** |  |  |  |  |  |  |  |  |
| No(reference) |  |  |  |  |  |  |  |  |
| Yes | 2.35(0.9-6.4) | 0.09 | 2.89(1.0-8.1) | P<0.05 | 2.12(1.3-3.5) | P<0.05 | 2.47(1.5-4.2) | P<0.05 |
| **Potentially Inappropriate Medicines** | | | | | | | | |
| No(reference) |  |  |  |  |  |  |  |  |
| Yes | 0.43(0.3-0.7) | P<0.05 | 0.41(0.2-0.7) | P<0.05 | 0.70(0.6-0.9) | P<0.05 | 0.63(0.5-0.8) | P<0.05 |
| **Charlson’s Comorbidity Index** | | | | | | | | |
| 0(reference) |  |  |  |  |  |  |  |  |
| ≥1 | 0.70(0.6-0.8) | P<0.05 | 0.70(0.6-0.8) | P<0.05 | 0.95(0.9-1.0) | 0.10 | 0.96(0.9-1.0) | 0.19 |

Table 2: Relationship between polypharmacy and hospitalization at one and five years expressed as HRs with 95% confidence interval

|  | **ONE YEAR** | | | | **FIVE YEARS** | | | |
| --- | --- | --- | --- | --- | --- | --- | --- | --- |
|  | **Univariate** | | **Multivariate** | | **Univariate** | | **Multivariate** | |
|  | **HR 95% CI** | **P value** | **HR 95% CI** | **P value** | **HR 95% CI** | **P value** | **HR 95% CI** | **P value** |
| **Polypharmacy** |  | | | | | | | |
| No(reference) |  | | | | | | | |
| Yes | 1.95(1.1-3.4) | P<0.05 | 2.47(1.4-4.3) | P<0.05 | 1.62(1.4-1.9) | P<0.05 | 1.49(1.3-1.7) | P<0.05 |
| **Age** | 1.10(1.1-1.2) | P<0.05 | 1.08(1.0-1.1) | P<0.05 | 1.04(1.0-1.1) | P<0.05 | 1.04(1.0-1.05) | P<0.05 |
| **Gender** |  | | | | | | | |
| Female(reference) | | | | | | | | |
| Male | 0.68(0.4-1.2) | 0.20 | 0.76(0.4-1.4) | 0.38 | 0.98(0.8-1.2) | 0.88 | 1.06(0.9-1.2) | 0.49 |
| **Previous Hospitalization** | | | | | | | | |
| No(reference) |  | | | | | | | |
| Yes | 2.33(1.4-3.9) | P<0.05 | 2.19(1.3-3.7) | P<0.05 | 1.7(1.5-1.9) | P<0.05 | 1.56(1.3-1.8) | P<0.05 |
| **Previous Falls** |  | | | | | | | |
| No(reference) |  |  |  |  |  |  |  |  |
| Yes | 2.41(0.7-7.7) | 0.14 | 3.14(0.9-10) | 0.06 | 1.52(0.9-2.3) | 0.05 | 1.39(0.9-2.1) | 0.13 |
| **Potentially Inappropriate Medicines** | | | | | | | | |
| No(reference) |  |  |  |  |  |  |  |  |
| Yes | 0.40(0.2-0.7) | P<0.05 | 0.40(0.2-0.6) | P<0.05 | 1.16(0.9-1.4) | 0.07 | 1.03(0.9-1.2) | 0.72 |
| **Charlson’s Comorbidity Index** | | | | | | | | |
| 0(reference) |  |  |  |  |  |  |  |  |
| ≥1 | 0.69(0.6-0.8) | P<0.05 | 0.72(0.6-0.8) | P<0.05 | 1.05(1.0-1.1) | P<0.05 | 1.01(0.9-1.1) | 0.46 |

Table 3: Relationship between Polypharmacy and falls at one and five years expressed as HRs with 95% confidence interval

|  | **ONE YEAR** | | | | **FIVE YEARS** | | | |
| --- | --- | --- | --- | --- | --- | --- | --- | --- |
|  | **Univariate** | | **Multivariate** | | **Univariate** | | **Multivariate** | |
|  | **HR 95% CI** | **P value** | **HR 95% CI** | **P value** | **HR 95% CI** | **P value** | **HR 95% CI** | **P value** |
| **Polypharmacy** |  | | | | | | | |
| No(reference) |  | | | | | | | |
| Yes | 0.38(0.04-3.7) | 0.41 | 0.37(0.03-4.0) | 0.41 | 1.64(1.0-2.6) | P<0.05 | 1.49(0.9-2.4) | 0.11 |
| **Age** | 1.19(1.0-1.4) | P<0.05 | 1.17(0.9-1.4) | 0.08 | 1.09(1.0-1.1) | P<0.05 | 1.09(1.0-1.1) | P<0.05 |
| **Gender** |  | | | | | | | |
| Female(reference) | | | | | | | | |
| Male | 0.59(0.06-5.7) | 0.65 | 0.77(0.07-7.8) | 0.82 | 0.78(0.4-1.3) | 0.34 | 0.97(0.6-1.6) | 0.91 |
| **Previous Hospitalization** | | | | | | | | |
| No(reference) |  | | | | | | | |
| Yes | 7.00(0.7-67) | 0.09 | 8.47(0.8-83) | 0.06 | 1.29(0.8-2.1) | 0.29 | 1.14(0.7-1.9) | 0.61 |
| **Previous Falls** |  | | | | | | | |
| No(reference) |  |  |  |  |  |  |  |  |
| Yes | omitted |  | omitted |  | 1.28(0.3-5.2) | 0.73 | 1.21(0.3-4.9) | 0.79 |
| **Potentially Inappropriate Medicines** | | | | | | | | |
| No(reference) |  |  |  |  |  |  |  |  |
| Yes | 0.38(0.05-2.7) | 0.34 | 0.48(0.06-3.8) | 0.49 | 1.21(0.7-2.1) | 0.51 | 1.04(0.6-1.8) | 0.89 |
| **Charlson’s Comorbidity Index** | | | | | | | | |
| 0(reference) |  |  |  |  |  |  |  |  |
| ≥1 | 0.79(0.4-1.5) | 0.47 | 0.93(0.5-1.7) | 0.82 | 1.01(0.9-1.1) | 0.84 | 0.97(0.9-1.1) | 0.71 |

Table 4: Relationship between polypharmacy and ADRs at one and five years expressed as HRs with 95% confidence interval

|  | **ONE YEAR** | | | | **FIVE YEARS** | | | |
| --- | --- | --- | --- | --- | --- | --- | --- | --- |
|  | **Univariate** | | **Multivariate** | | **Univariate** | | **Multivariate** | |
|  | **HR 95% CI** | **P value** | **HR 95% CI** | **P value** | **HR 95% CI** | **P value** | **HR 95% CI** | **P value** |
| **Polypharmacy** |  | | | | | | | |
| No(reference) |  | | | | | | | |
| Yes | omitted |  | omitted |  | 1.39(0.8-2.4) | 0.29 | 0.97(0.5-1.8) | 0.93 |
| **Age** | 1.74(1.1-2.8) | P<0.05 | omitted |  | 1.07(1.0-1.1) | P<0.05 | 1.07(1.0-1.1) | P<0.05 |
| **Gender** |  | | | | | | | |
| Female(reference) | | | | | | | | |
| Male | omitted |  | omitted |  | 0.90(0.5-1.6) | 0.73 | 0.98(0.5-1.8) | 0.94 |
| **Previous Hospitalization** | | | | | | | | |
| No(reference) |  | | | | | | | |
| Yes | omitted |  | omitted |  | 2.48(1.4-4.4) | P<0.05 | 2.21(1.2-3.9) | P<0.05 |
| **Previous Falls** |  | | | | | | | |
| No(reference) |  |  |  |  |  |  |  |  |
| Yes | omitted |  | omitted |  | 3.26(1.0-10.5) | P<0.05 | 3.22(0.9-10.5) | 0.05 |
| **Potentially Inappropriate Medicines** | | | | | | | | |
| No(reference) |  |  |  |  |  |  |  |  |
| Yes | omitted |  | omitted |  | 1.71(0.8-3.6) | 0.16 | 1.34(0.6-2.9) | 0.45 |
| **Charlson’s Comorbidity Index** | | | | | | | | |
| 0(reference) |  |  |  |  |  |  |  |  |
| ≥1 | 1.18(0.5-2.7) | 0.68 | omitted |  | 1.17(1.0 -1.3) | P<0.05 | 1.14(1.0-1.3) | P<0.05 |

**Subgroup analysis**

Table 5: Relationship between polypharmacy and mortality for men

Table 6: Relationship between polypharmacy and mortality for women

Table 7: Relationship between polypharmacy and hospitalization for men

Table 8: Relationship between polypharmacy and hospitalization for women

Table 9: Relationship between polypharmacy and mortality for participants aged 75-85 years

Table 10: Relationship between polypharmacy and mortality for participants aged 86years and over

Table 11: Relationship between polypharmacy and hospitalization for participants aged 75-85 years

Table 12: Relationship between polypharmacy and hospitalization for participants aged 86years and over

Table 13: Relationship between polypharmacy and mortality for participants with 1-5 potentially inappropriate medicines

Table 14: Relationship between polypharmacy and mortality for participants with six or more potentially inappropriate medicines

Table 15: Relationship between polypharmacy and hospitalization for participants with 1- 5 potentially inappropriate medicines

Table 16: Relationship between polypharmacy and hospitalization for participants with six or more potentially inappropriate medicines

Table 17: Relationship between polypharmacy and mortality in participants with 1-2 morbidities

Table 18: Relationship between polypharmacy and mortality in participants with 3-6 morbidities

Table 19: Relationship between polypharmacy and hospitalisation in participants with 1-2 morbidities

Table 20: Relationship between polypharmacy and hospitalisation in participants with 3-6 morbidities

**Interactions**

**Table 21: Effect of Interaction between polypharmacy and age groups on mortality**

**Table 22: Effect of Interaction between polypharmacy and gender groups on mortality**

**Table 23: Effect of Interaction between polypharmacy and PIM groups on mortality**

**Table 24: Effect of Interaction between polypharmacy and comorbidity groups on mortality**

**Table 25: Effect of Interaction between polypharmacy and age groups on hospitalization**

**Table 25: Effect of Interaction between polypharmacy and gender groups on hospitalization**

**Table 26: Effect of Interaction between polypharmacy and PIM groups on hospitalization**

**Table 27: Effect of Interaction between polypharmacy and comorbidity groups on hospitalization**
